# Crystal Structure of the Vaccinia Virus DNA Polymerase Holoenzyme Subunit D4 in Complex with the A20 N-Terminal Domain

**DOI:** 10.1371/journal.ppat.1003978

**Published:** 2014-03-06

**Authors:** Céline Contesto-Richefeu, Nicolas Tarbouriech, Xavier Brazzolotto, Stéphane Betzi, Xavier Morelli, Wim P. Burmeister, Frédéric Iseni

**Affiliations:** 1 Unité de Virologie, Institut de Recherche Biomédicale des Armées, Brétigny-sur-Orge, France; 2 Université Grenoble Alpes, UVHCI, Grenoble, France; 3 CNRS, UVHCI, Grenoble, France; 4 Unit for Virus Host-Cell Interactions, UMI 3265, Université Grenoble Alpes-EMBL-CNRS, Grenoble, France; 5 Département de Toxicologie et Risque Chimique, Institut de Recherche Biomédicale des Armées, Brétigny-sur-Orge, France; 6 Centre de Recherche en Cancérologie de Marseille (CRCM), CNRS UMR 7258, INSERM U 1068, Institut Paoli-Calmettes & Aix-Marseille Universités, Marseille, France; National Institute of Allergy and Infectious Diseases, United States of America

## Abstract

Vaccinia virus polymerase holoenzyme is composed of the DNA polymerase E9, the uracil-DNA glycosylase D4 and A20, a protein with no known enzymatic activity. The D4/A20 heterodimer is the DNA polymerase co-factor whose function is essential for processive DNA synthesis. Genetic and biochemical data have established that residues located in the N-terminus of A20 are critical for binding to D4. However, no information regarding the residues of D4 involved in A20 binding is yet available. We expressed and purified the complex formed by D4 and the first 50 amino acids of A20 (D4/A20_1–50_). We showed that whereas D4 forms homodimers in solution when expressed alone, D4/A20_1–50_ clearly behaves as a heterodimer. The crystal structure of D4/A20_1–50_ solved at 1.85 Å resolution reveals that the D4/A20 interface (including residues 167 to 180 and 191 to 206 of D4) partially overlaps the previously described D4/D4 dimer interface. A20_1–50_ binding to D4 is mediated by an α-helical domain with important leucine residues located at the very N-terminal end of A20 and a second stretch of residues containing Trp43 involved in stacking interactions with Arg167 and Pro173 of D4. Point mutations of the latter residues disturb D4/A20_1–50_ formation and reduce significantly thermal stability of the complex. Interestingly, small molecule docking with anti-poxvirus inhibitors selected to interfere with D4/A20 binding could reproduce several key features of the D4/A20_1–50_ interaction. Finally, we propose a model of D4/A20_1–50_ in complex with DNA and discuss a number of mutants described in the literature, which affect DNA synthesis. Overall, our data give new insights into the assembly of the poxvirus DNA polymerase cofactor and may be useful for the design and rational improvement of antivirals targeting the D4/A20 interface.

## Author Summary

Vaccinia virus is the prototype of the *orthopoxvirus* genus which includes other pathogens infecting humans and variola virus which was eradicated in the late 70's. Vaccinia virus DNA synthesis relies on three proteins: these are E9, the DNA polymerase bound to its heterodimeric cofactor D4/A20. To date, the molecular mechanism involved in poxvirus DNA replication remains poorly understood. Here, we present the high-resolution crystal structure of a complex formed by D4 and the first 50 residues of A20 (A20_1–50_) that are necessary and sufficient for binding. The structure of D4/A20_1–50_ reveals the contact surface engaged in the D4/A20 interaction in great detail. Interestingly, we could show that known small molecule inhibitors of vaccinia virus DNA synthesis selected for their ability to interfere with the D4/A20 interface could be docked onto the D4 surface where they mimic several aspects of the interacting A20 molecule. Finally, we present a model of D4/A20 in complex with DNA that allows us to discuss the role of mutations affecting the D4/A20 cofactor. Altogether, our structure gives new insights into the assembly of the vaccinia virus DNA polymerase cofactor and will be useful for the design of new antiviral compounds targeting the D4/A20 interaction.

## Introduction

The well-studied vaccinia virus (VACV) belongs to the *orthopoxvirus* genus of the family *poxviridae*. The O*rthopoxvirus* genus also comprises well-known pathogens such as monkeypox virus and cowpox virus (which can be transmitted to humans) as well as the most virulent member variola virus. Unlike other DNA viruses, orthopoxviruses replicate entirely in the cytoplasm of the infected host-cell. Viral genome synthesis takes place in perinuclear foci called viral factories and is thought to depend almost exclusively on virally encoded-proteins. Four of these proteins, presumably positioned at the replication fork, were shown to be essential for DNA synthesis [1]. For VACV these are: E9, the catalytic subunit of the DNA polymerase; D5, a DNA-independent nucleoside triphosphatase which contains a putative helicase domain [2] and primase activity [3]; D4, a uracil-DNA glycosylase (UDG) [4] and A20, a central component linking E9 and D4 [5], [6] and interacting with D5 [7], [8].

The catalytic DNA polymerase E9 alone is distributive under physiological conditions [9]. However, it becomes highly processive when bound to its heterodimeric co-factor D4/A20, forming the processive DNA polymerase holoenzyme [10]. The presence of a DNA repair protein (D4) as necessary component of the VACV replication machinery is intriguing and unusual for DNA viruses. UDGs encoded by several herpes viruses have been characterized so far and in contrast to poxviruses these were shown to behave as accessory proteins rather than essential factors during DNA synthesis. Indeed, when deletion mutants lacking UDG were built, the recombinant herpes viruses were replication-competent and viable [11]–[14]. The function of UDG is to prevent accumulations of uracil bases in DNA molecules due to misincorporation of dUTP or spontaneous cytosine deamination by excision of the uracil moiety and initiation of the base-excision repair pathway [15]. Another striking feature of D4 as component of the processivity factor is that while its presence is crucial for DNA replication, *i.e* knock-out mutants lacking D4 are not viable [16], [17], its glycosylase activity is dispensable [18]. These observations raised the question how D4/A20 confers processivity to E9. Data obtained from the Traktman group provided evidence in favor of a model in which the intrinsic DNA scanning activity of D4 stimulates long-chain DNA synthesis by E9 [5]. Indeed, the process of uracil search by UDG implies random diffusion on the DNA molecule in a sequence independent manner (DNA hopping and sliding) [19]. Then, the enzyme kinks and compresses the duplex DNA backbone in order to extrude each base to examine its identity (so called “pinch-push-pull” mechanism) [20]–[22]. Only uracil bases enter the uracil binding pocket allowing the hydrolysis of the glycosyl bond. Since D4 does not interact directly with E9 it is proposed that A20 forms the link between the UDG and the DNA polymerase catalytic subunit E9 [10]. In agreement with this model, our recent low-resolution structure of VACV D4/A20/E9 complex established a 150 Å separation between the polymerase active site of E9 and the DNA-binding site of D4 while the elongated A20 protein connects E9 and D4 [6].

Various mutations affecting D4 and A20 proteins have been described in the literature. These mutations were obtained from temperature-sensitive viruses isolated after mutagenesis [5], [10], [23], [24] or were engineered into the D4R and A20R genes by site-directed mutagenesis [25]–[28]. These mutants are of great importance for mechanistic insights into the VACV DNA synthesis. However, when the mutant proteins are used in a reconstituted *in vitro* replication system, it is often difficult to clearly explain the observed phenotype [28]. This is partially due to the lack of a high-resolution structure of the VACV replication complex allowing positioning of the mutations. So far, our knowledge about the assembly of the D4/A20/E9 complex remains very imprecise. While the N-terminal 25 amino acids of A20 are important for D4 binding [7], the region of D4 involved in A20 interaction is still unknown and no data are yet available regarding the A20/E9 interaction.

Soluble VACV D4 has been successfully expressed in bacteria. The concentrated recombinant protein forms dimers in solution and the dimeric assembly is maintained when the protein is crystallized [29]. Whether or not an oligomeric form of D4 is necessary for its activity within the VACV polymerase holoenzyme is still a matter of debate as another study suggests that virally expressed D4 would rather behave as a monomer [5]. Our low-resolution structure of D4/A20/E9 complex showed a 1∶1∶1 stoichiometry, consistent with D4 being in a monomeric state in the complex [6].

In order to further investigate the molecular structure of the VACV DNA replication machinery, we decided to study specifically the D4/A20 dimer interface. As mentioned previously, the first 25 residues of A20 have been shown to be the minimal binding region required for interaction with D4, however optimal binding was observed with the first 50 amino acids [7]. In this report, we present the co-expression and purification of VACV D4 bound to the first 50 residues of A20 (D4/A20_1–50_). We showed that whereas D4 alone most likely forms homodimers in solution, D4/A20_1–50_ clearly behaves as a heterodimer. Determination of the high-resolution structure of D4/A20_1–50_ reveals that the A20_1–50_ interaction on the D4 surface partially overlaps the D4/D4 dimer interface. A structure-based site-directed mutagenesis study allowed the identification of new residues that modulate the D4/A20 interaction. These residues could potentially be the target of poxvirus inhibitors known to interfere with the D4/A20 interaction [30]. Finally, we have proposed a model of D4/A20_1–50_ in complex with DNA that allows us to discuss the observed phenotype of several mutants described in the literature [24], [28].

## Results

### Recombinant vaccinia virus His-D4/A20_1–50_ forms a heterodimeric complex

**Figure 1:**
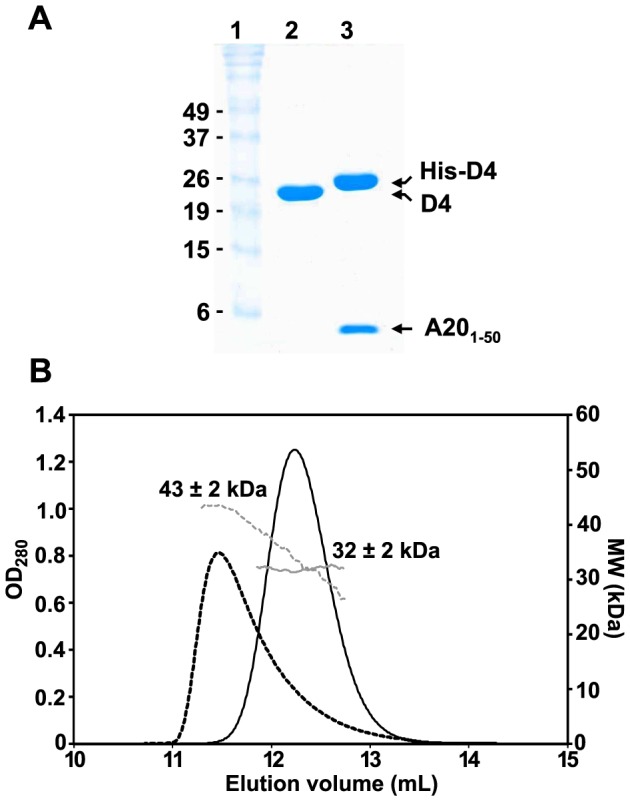
Oligomeric state of D4 and His-D4/A20_1–50_. (A) 15% SDS-PAGE analysis of purified D4 and His-D4/A20_1–50_ complex. Lane 1: Molecular weight standards. Lane 2: D4 (∼10 µg). Lane 3: His-D4/A20_1–50_ (∼10 µg). Proteins were stained with InstantBlue (Expedeon). (B) Analysis of D4 and His-D4/A20_1–50_ by SEC-MALLS. D4 elutes at 11.5 mL (black dashed line). The molecular mass of 43±2 kDa at the maximum peak height decreases down to ∼28 kDa suggesting that a monomer/dimer equilibrium of D4 (calculated molecular mass of 25.4 kDa) exists in solution (grey dashed line). His-D4/A20_1–50_ (calculated molecular mass of 32.4 kDa) elutes as a symmetrical peak at 12.2 mL (black line) with a constant molecular mass of 32±2 kDa (grey line) suggesting a 1∶1 stoichiometry for the complex.

The N-terminal domain of A20 has been shown to be necessary to interact with D4 [7], which has a tendency to dimerize when over-expressed as a recombinant protein in bacteria [29]. In order to determine the oligomeric state of a complex formed by D4 and A20_1–50_, we co-expressed in bacteria N-terminal His-tagged D4 together with A20_1–50_ fused to the C-terminus of the maltose binding protein (MBP), downstream of a TEV protease cleavage site. In parallel, we also expressed a recombinant form of D4 containing a TEV-cleavable vector-derived N-terminal hexa-histidine tag. Lysates containing either His-D4 alone or His-D4 and MBP-A20_1–50_ were loaded onto a HisTrap HP column and eluted with imidazole. After TEV protease treatment, proteins were passed again over the nickel column and further purified by size exclusion chromatography. Purified recombinant D4 without His-tag migrates in SDS-PAGE according to its calculated molecular mass of 25.4 kDa (Figure 1A, lane 2). A20_1–50_ (5.7 kDa) and His-D4 (26.7 kDa) co-eluted through the various purification steps (Figure 1A, lane 3), demonstrating complex formation between the two proteins.

In order to obtain the absolute molecular weight of recombinant D4 and His-D4/A20_1–50_, SEC-MALLS experiments were performed (Figure 1B). D4 elutes from the gel filtration column at 11.5 mL as a broad and tailed peak. At the maximum peak height, a molecular mass of 43±2 kDa was determined. However, the measured molecular mass decreases throughout the tail of the chromatogram down to about 28 kDa. Thus, our results indicated that D4 is not monodisperse in solution (M_w_/M_n_ = 1.011) and suggested that at high concentration (10 mg.mL^−1^) a fast monomer-dimer equilibrium is observed (expected molecular mass: 25.4 kDa and 50.8 kDa, respectively). In contrast, using the same experimental conditions, His-D4/A20_1–50_ elutes as a sharp and symmetrical peak at 12.2 mL. The molecular mass of 32±2 kDa determined by MALLS is consistent with the formation of a 1∶1 heterodimeric complex (Figure 1B, theoretical molecular mass: 32.4 kDa). In addition, the ratio M_w_/M_n_ = 1.000 obtained from the MALLS experiment indicated that the isolated complex is monodisperse in solution. Taken together, our results are consistent with the ability of D4 to form dimers in solution [29], however, when co-expressed with A20_1–50_, His-D4/A20_1–50_ clearly forms a tight heterodimeric complex.

### Crystal structure of the His-D4/A20_1–50_ complex

His-D4/A20_1–50_-WT as well as His-D4/A20_1–50_-T2A (carrying a Thr to Ala point mutation at position 2) crystallized at pH 8.7 in space group P3_1_21 with unit-cell parameters a = b = 92.6, c = 145.9 Å (Table 1). Crystals measuring about 200×200×200 µm were obtained. Diffraction data were collected up to 1.85 Å for His-D4/A20_1–50_-T2A and to 2.2 Å for the wild type complex (Table 1).

**Table 1:** Data collection and refinement statistics.

	D4/A20_1–50_T2A	D4/A20_1–50_WT
**Data collection and processing**		
Beamline	ESRF ID23-1	ESRF ID14-4
Space group	P3_1_2	P3_1_2
Unit-cell parameters		
a, b, c (Å)	92.62, 92.62, 145.86	92.98, 92.98, 145.71
α, β, γ (°)	90, 90, 120	90, 90, 120
Wavelength (Å)	0.9763	0.9394
Resolution (Å)	48.62-1.85 (1.95-1.85)	54.02-2.20 (2.32-2.20)
No. of observed reflections	438637 (62038)	199023 (28671)
No. of unique reflections	61957 (8912)	37537
Completeness (%)	99.9 (99.9)	99.7 (99.9)
Multiplicity	7.1 (7.0)	5.3 (5.3)
Mean *I/σ(I)*	15.8 (3.5)	13.2 (4.0)
R*_merge_*	0.072 (0.542)	0.075 (0.516)
**Refinement and model composition:**		
*R*work/*R*free	0.186 (0.27)/0.230 (0.30)	0.196 (0.30)/0.252 (0.36)
No. residues		
Protein	542	543
Water	341	154
R.m.s. deviations		
Bond lengths (Å)	0.021	0.018
Bond angles (°)	2.207	2.107
Average B-factor (Å^2^)		
Protein	17.8	20.8
Water	37.7	44.9

**Figure 2:**
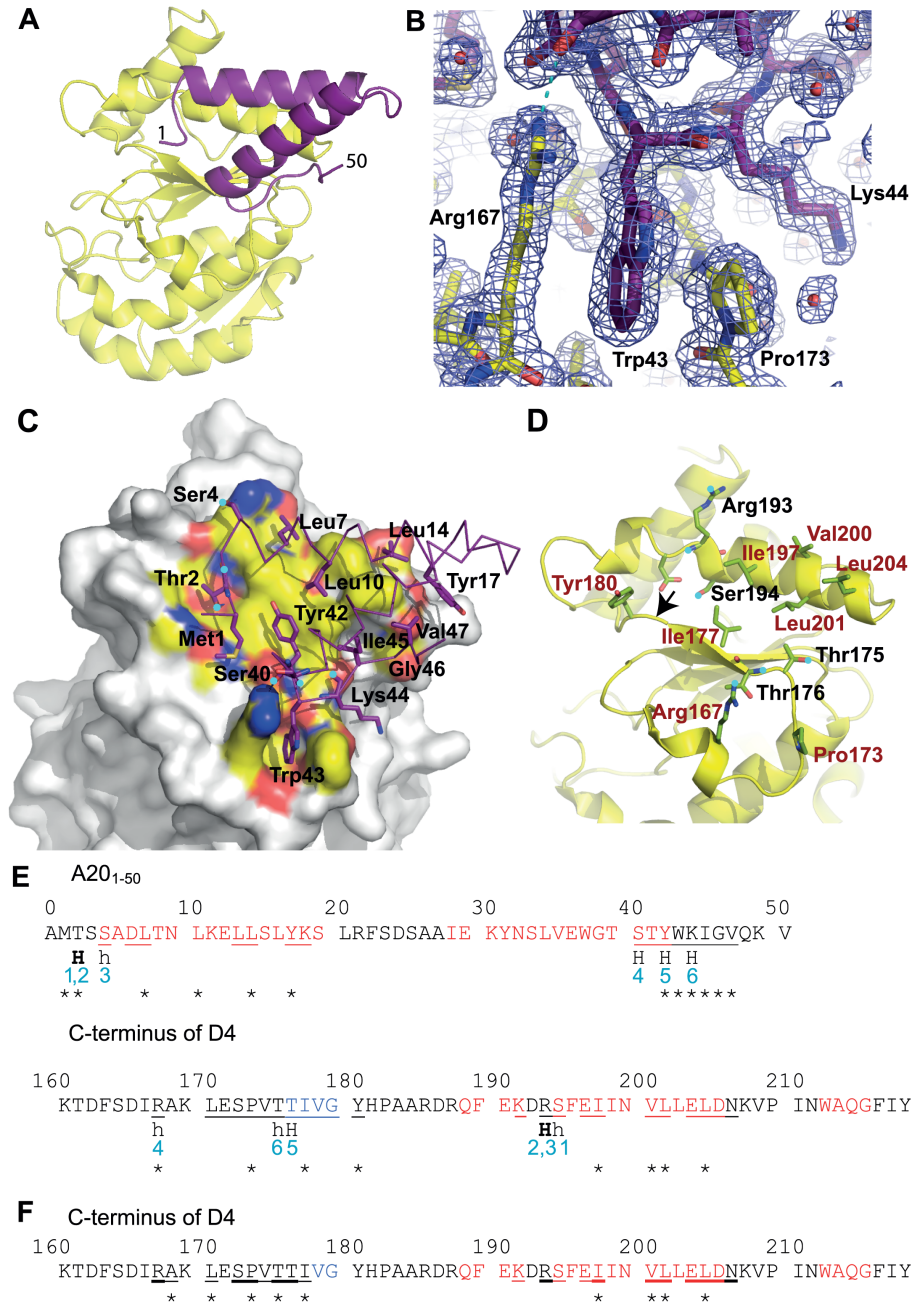
Crystal structure of His-D4/A20_1–50_ complex. D4 is shown with yellow carbon atoms, A20_1–50_ in violet. Oxygen atoms are colored in red, nitrogen atoms in blue. Panels (A), (B), (C) and (D) use the same orientation. (A) View of the complex in cartoon representation. (B) SigmaA-weighted 2Fo-Fc electron density map of the refined model of His-D4/A20_1–50_-T2A at 1.85 Å resolution contoured at 1σ in the vicinity of Trp43 of A20 and Arg167 and Pro173 of D4. A cyan dotted line indicates a hydrogen bond. (C) Surface representation of D4, where only atoms within 4.5 Å of A20 are colored, with the principal side chains of A20_1–50_ forming the interface (see panel E) shown in stick representation. When contacts involve main chain atoms, the main chain of the corresponding residues is shown. Hydrogen bond donors and acceptors are highlighted with cyan dots. (D) The principal residues of D4 which are involved in the contact with A20_1–50_ are shown in light green; residues involved in hydrophobic contacts are printed in brown. Cyan dots mark hydrogen bond acceptors and donors. The arrow indicates the position of the Gly179Arg mutation described in [10]. (E) The D4/A20 contacts. Residues involved in α-helices are printed in red, the ones involved in β-sheets in blue. Residues with some of their surface buried in the contact are underlined. Residues providing hydrogen bond donors or acceptors for inter-subunit hydrogen bonds are marked «H». H-bonds in capitals involve main chain atoms; in small letters side chain atoms; H-bonds in bold indicate residues forming H-bonds with both side chain and main chain atoms. H-bonds are numbered in blue. Stars indicate the residues which contribute with more than 0.2 kcal⋅mol^−1^ to the interaction according to the the PISA server [31] used for analysis of the interface. (F) The D4/D4 contacts. Residues which are involved in the dimer contact on both monomers are underlined in bold; residues interacting asymmetrically are simply underlined. The analysis was performed with the pdb entry 4DOF, chain A/B.

The structure solved by molecular replacement using a D4 monomer from Schormann *et al*. (pdb: 2OWQ, [29]) is shown in Figure 2A. Two His-D4/A20_1–50_ complexes are present per asymmetric unit. All residues of A20_1–50_ could be modelled and refined (Figure 2B) including an additional N-terminal Ala residue (Ala0) that remains after the TEV cleavage step. The structure of D4 in complex with the A20 N-terminus is identical to the revised model of free D4 (pdb entry 4DOF, chain A, rms 0.30 Å) when residues 164 to 174 are excluded from the superposition. The conformation of residues 164 to 174 shows a large variability in the different available homodimer structures but these residues are well ordered in our complex structure.

The structure of A20_1–50_-WT is almost identical to the one of A20_1–50_-T2A: the effect of the mutation is strictly local and affects only the mutated residue. A20_1–50_ forms two α-helices that are packed against each other. The remaining residues connecting the two helices and the ones located at the extremities of the peptide do not show any secondary structure (Figures 2A and 2E). The loop connecting the two helices (residues 20 to 27), which is not involved in the D4/A20_1–50_ interface shows some variability when the two complexes of the asymmetric unit are compared (data not shown). When this connector region is excluded from a superposition, the Cα atoms of the two complexes present in the asymmetric unit can be superimposed with 0.29 Å rms deviation.

### Analysis of the D4/A20_1–50_ interface

Both proteins form an extensive contact surface (1890 Å^2^ of buried surface, Figures 2C and 2D). The contact is strikingly flat with the exception of the prominent residue Trp43 of A20 sandwiched between Pro173 and Arg167 of D4 (Figure 2B). The contact surface is formed essentially by hydrophobic residues, but 6 hydrogen bonds confer the required specificity to the interaction (Figures 2C, 2D and 2E). In A20, two stretches of residues form the contact surface: residues 1 to 14 are located in the loop structure at the extremity of the fragment and within the 1^st^ helix; residues 40 to 47 are located at the end of the 2^nd^ helix and in the following loop structure. Likewise, in D4 two stretches of residues form the contact surface: residues 167–180 and 191–206 that are present within loop structures, a β-strand and a α-helix (Figures 2D and 2E).

On the A20 side of the interface the main contributing residues are Met1, Thr2, Leu7, Leu10, Leu14, Tyr17, Tyr42, Trp43, Lys44, Ile45, Gly46 and Val47, each contributing for more than 0.2 kcal.mol^−1^ to the binding as estimated with PISA [31] (Figures 2C and 2E). Additional residues are involved in hydrogen bonds: Ser4 and Ser40. A few more residues are located in the contact surface but contribute only marginally to binding. On the D4 side of the interface, Arg167 and Pro173 contribute to the interface whereas Ile197, Val200, Leu201 and Leu204 are the main hydrophobic contributors and Thr175, Thr176, Arg193 and Ser194 are the partners involved in hydrogen bond formation (Figures 2D and 2E).

### Point mutations at the D4/A20 interface affect complex formation and stability

**Figure 3:**
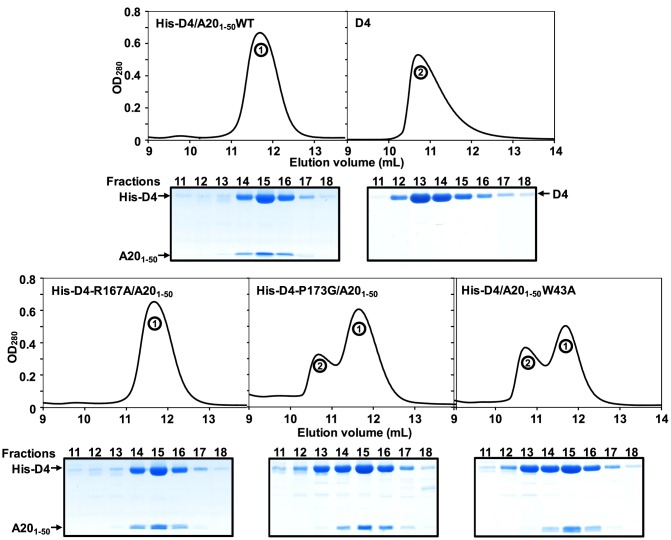
Point mutations at the D4/A20_1–50_ interface affect complex formation. D4, His-D4/A20_1–50_WT and His-D4/A20_1–50_ mutants were purified as described in the Materials and Methods section. Protein elution profiles after the last purification step (i.e. size exclusion chromatography) are presented. 15% SDS-PAGE analysis of the peak fractions (11 to 18) is aligned with each chromatogram. Proteins were stained with InstantBlue (Expedeon). Migration of D4, His-D4 and A20_1–50_ is indicated. Peak 1 and peak 2 are labelled.

The crystal structure of His-D4/A20_1–50_ highlights the importance of several Leu residues (Leu7, Leu10 and Leu14) of A20 for interaction with D4 (Figures 2C and 2E). In accordance with our findings it was previously shown that Leu to Ala mutation affecting these specific amino acids interfered with D4/A20 complex formation [5]. We then wanted to determine the contribution of the previously unknown contact generated by Trp43 of A20 that is involved in stacking interactions with Arg167 and Pro173 of D4 (Figure 2B). For this purpose, point mutations were introduced into the pETDuet-D4R-A20R_1–50_WT construct in order to produce three mutants: His-D4/A20_1–50_W43A, His-D4-R167A/A20_1–50_ and His-D4-P173G/A20R_1–50_. Mutant proteins were expressed and purified as their wild-type counterparts. As a control, D4 expressed alone was also purified. Figure 3 shows the elution profiles of D4, His-D4/A20_1–50_WT and His-D4/A20_1–50_ mutants after size exclusion chromatography: His-D4/A20_1–50_WT elutes (as previously observed, Figure 1B) as a single peak (Figure 3, top left panel, peak 1). A similar elution profile is observed for the mutant His-D4-R167A/A20_1–50_ (Figure 3, bottom left panel). However, chromatograms of His-D4-P173G/A20R_1–50_ and His-D4/A20_1–50_W43A mutants (bottom middle and right panels) showed the presence of two distinct peaks. Proteins from peak 1 present a similar elution volume (11.7 mL) than the one observed for His-D4/A20_1–50_WT and His-D4-R167A/A20_1–50_, while proteins from the second peak have a lower elution volume of 10.7 mL which is identical to the one of D4 homodimer (Figure 3, top right panel, peak 2). 15% SDS-PAGE fraction analysis of the peaks is also presented in Figure 3. Fractions from peak 1 show the typical protein pattern obtained previously when His-D4 and A20_1–50_ form the heterodimeric complex (Figure 1A). However, fractions from peak 2 clearly show an excess of D4 and little or no A20_1–50_ peptide (Figure 3, see fractions 12 and 13). The shorter retention time of D4 from peak 2 (compared to His-D4/A20_1–50_ from peak 1) is consistent with the presence of D4 homodimers in these fractions. Thus, our results demonstrated that A20 Trp43 and D4 Pro173 are critical amino acids at the D4/A20_1–50_ interface and that mutations of these residues interfere with D4/A20_1–50_ complex formation resulting in D4 homodimer assembly. In contrast, D4 Arg167 does not seem to be essential for D4/A20_1–50_ complex formation.

**Figure 4:**
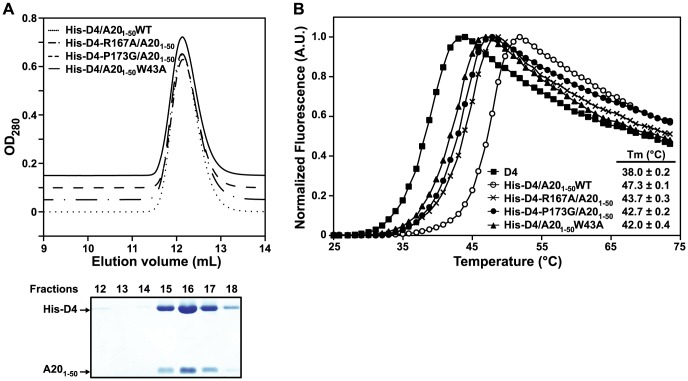
Point mutations at the D4/A20_1–50_ interface affect complex stability. (A) Fractions from Peak 1 after purification of WT and His-D4/A20_1–50_ mutants (see Figure 3) were pooled and loaded again onto a gel filtration column. Chromatograms of WT and His-D4/A20_1–50_ mutants are superimposed with 0.05 OD offset. A typical 15% SDS-PAGE analysis of the peak fractions (12 to 18) is shown below (in this case His-D4/A20_1–50_W43A). Proteins were stained with InstantBlue (Expedeon). Migration of His-D4 and A20_1–50_ is indicated. (B) A representative thermal shift experiment is shown. For D4 and each His-D4/A20_1–50_ complex calculated *T_m_* values obtained from three independent experiments are given together with their standard deviation.

To study further the behaviour of His-D4/A20_1–50_ mutants and to compare them with the WT complex, fractions of peak 1 were pooled and resubmitted to size exclusion chromatography (Figure 4A). WT and mutant complexes elute as a single and sharp peak during this second chromatographic step. It is noteworthy that whatever the mutant, no peak with shorter retention time (see peak 2 in Figure 3) was observed during this second gel-filtration chromatography and SDS-PAGE analysis of the peak fractions showed co-elution of His-D4 and A20_1–50_ (Figure 4A). As no D4 homodimer is formed dynamically, this indicates that once assembled, the His-D4/A20_1–50_ complex is stable even in presence of mutations at the interface.

Thermal shift assays were performed with these re-purified complexes to monitor the thermal stability of the different mutants compared to the WT complex (Figure 4B). A *T_m_* of 47.3±0.1°C was determined for the His-D4/A20_1–50_WT complex. All three mutant complexes have about 4 to 5°C lower *T_m_*, indicating that they are less stable than the WT. Thermal stability of His-D4 was also assayed in this experiment. A *Tm* of 38.0±0.2°C was determined and is in agreement with the result previously obtained by Nuth *et al.* (*Tm* of 38.4±1.6°C) [32]. The key role of A20 Trp43, D4 Arg167 and D4 Pro173 in the D4/A20_1–50_ interaction is reinforced by the thermal stability data.

## Discussion

It has been demonstrated that the complex formed by the VACV D4 and A20 proteins is essential to convert the distributive DNA polymerase E9 into a processive mode [5], [10]. Yet, much work remains to be done to understand the molecular mechanisms driving D4/A20 assembly and how it stimulates long-chain DNA synthesis. A first glimpse into the complex structure was obtained from the study of Schormann, *et al*. which has shown that bacterially expressed VACV D4 was found to be dimeric in solution and crystallized as a dimer [29]. The dimerization of D4 was intriguing since UDGs from different organisms are structurally well conserved and known to be small monomeric enzymes that do not require co-factors or even divalent cations for activity [33]. Additional biochemical and structural studies of the D4/A20 complex did not favor the model in which D4 functions as a dimer but rather suggested that within the DNA polymerase holoenzyme D4 is in a monomeric state [5], [6]. The data presented in this report strengthen this last model and explain at the molecular level how A20 prevents D4/D4 dimerization by binding to D4. The molecular mass ranging from 43 to 28 kDa obtained for D4 in the SEC-MALLS experiment is consistent with the protein existing as a mixture of monomer/dimer in solution, with a relatively large dissociation constant and fast kinetics. In contrast, when co-expressed with its partner A20_1–50_, a D4/A20_1–50_ complex is formed with a 1∶1 stoichiometry (molecular mass of 32 kDa, Figure 1).

**Figure 5:**
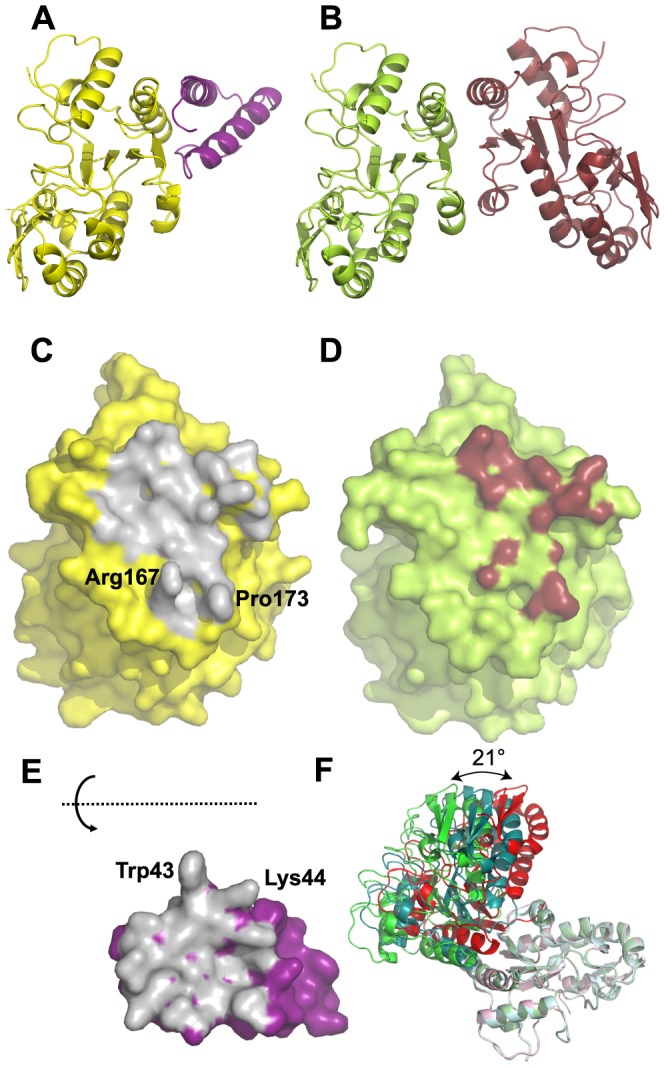
Comparison of D4/A20_1–50_ and D4/D4 interfaces. (A) Cartoon representation of the D4 (yellow)/A20_1–50_ (magenta) complex. (B) Cartoon representation of the D4 dimer (pdb entry 4DOF chain A in light green and chain B in brown). (C) Surface representation of D4 in the His-D4/A20_1–50_ complex structure. The atoms in contact with A20_1–50_ (meaning closer than 4.5 Å to atoms of A20_1–50_) are colored in grey. (D) Surface representation of D4 (pdb entry 4DOF, chain A) in the context of the D4 dimer structure. Atoms of D4 involved in the dimer contact are shown in brown (distance <4.5 Å). (E) Surface representation of A20_1–50_. The atoms in contact with D4 (distance <4.5 Å) are colored in grey. A20_1–50_ has been turned by 180° around a horizontal axis. (F) Three representative D4/D4 dimer structures present in the pdb differing in the relative orientation of the two subunits are shown. The A chains of the dimers have been superposed and are shown in light colors. Chain B from pdb entry 4DOF represents one class and is shown in green; the chain of the crystallographic dimer from pdb entry 4DOG which represents another class of orientations is shown in red, chain B from the dimer in pdb entry 2OWR with an intermediate orientation is shown in turquoise.

Comparison of both D4/D4 and His-D4/A20_1–50_ crystal structures indicates that the contact surfaces of D4 or A20_1–50_ on D4 are overlapping (Figures 5A to 5D) and illustrates the difference between a specific interaction (His-D4/A20_1–50_) and a non-specific one (D4/D4) (Figures 5C to 5E). In both interactions, most of the change in the Gibbs free energy (ΔG) upon binding is contributed by hydrophobic contacts. As the D4/A20_1–50_ interaction surface is strikingly flat, additional interactions have to be provided in order to define the relative orientation of the two partners and to ensure specificity. This is achieved by the 6 hydrogen bonds within the interface (Figure 2C, 2D and 2E). In addition to the flat interaction surface, the D4/A20 interaction uses steric complementarity forming a tongue and groove connection involving residues Arg167 and Pro173 on D4 (the “groove”) and Trp43 on A20 (the “tongue”) (Figure 2B).

In contrast, the less than perfect match of the two binding surfaces in the D4 dimer is obvious, first from the reduction of the buried surface area (for example 1030 Å^2^ for the A/B dimer of pdb entry 4DOF vs. 1890 Å^2^ for the D4/A20_1–50_ interface), which matches the visually much less pronounced contacts and a lesser surface complementarity (Figure 5C to 5E). Strikingly, no hydrogen bonds are involved in the contact (Figure 2F). The poor definition of the relative orientation of the two molecules in the D4/D4 dimer becomes obvious when the 9 available different crystallographic dimer structures are compared. The relative orientation of the subunits varies by 21° (Figure 5F). The dimers from pdb entry 4DOG, 2OWQ and 3NT7 cluster in one group, those from 4DOF together with 3 of the dimers from 2OWR in a second group. Finally, one dimer from pdb entry 2OWR (chains A and B) adopts an intermediate structure showing a rotation of 14° compared to the model from 4DOG (Figure 5F). Last but not least, the homodimer structures show variable conformations and a poor definition of two stretches of residues, 164–174 and 182–195, which contribute to the D4/D4 dimer contact. Overall, the results presented herein indicate that D4 is able to dimerize when over-expressed alone but in the presence of A20_1–50_, D4/D4 interactions are prevented and the formation of a stable heterodimeric D4/A20_1–50_ complex is favored. Thus during the course of the VACV infection the D4/A20 heterodimer forms the processivity factor of the DNA polymerase E9 and the D4 dimerization observed *in vitro* is likely to be an artifact.

Previous results from the Moss group showed that the minimal binding region necessary to bind to D4 resided within the N-terminal 25 amino acids of A20, although full binding was only observed when D4 was expressed together with the first 50 residues of A20 [7]. This suggested that additional residues located between amino acids 25 and 50 might be important for the D4/A20 interaction, a result fully confirmed by the structure of the D4/A20 interface. More recently, the highly conserved leucine residues within the first 25 amino acids of A20 were shown to be critical for the interaction [5]. Indeed, our crystal structure confirmed that Leu7, Leu10 and Leu14 played a key role in the D4/A20 interaction, whereas Leu13 and Leu16 rather form the hydrophobic core of the A20 fragment and are part of the contact between the two helices. Most importantly, the structure identifies a second contact in the D4/A20 complex, involving A20 Trp43 stacked between D4 Arg167 and Pro173. To determine if these residues are important for D4/A20 binding, we have mutated them individually and showed that each of these mutations had a significant negative effect on complex formation and stability. Mutations of the residues forming the tongue and groove interaction all lead to a reduced complex stability in a thermal shift assay and to formation of D4 homodimers for the mutants D4 Pro173Gly and A20 Trp43Ala. The effect of these mutations underlines the importance of this second binding site involving residues outside the initially identified residues 1–25 of A20. Thus, the results from Ishii *et al.* together with the data presented here strongly indicated that all the determinants for D4 binding are located within the first 50 residues of A20 [7].

**Figure 6:**
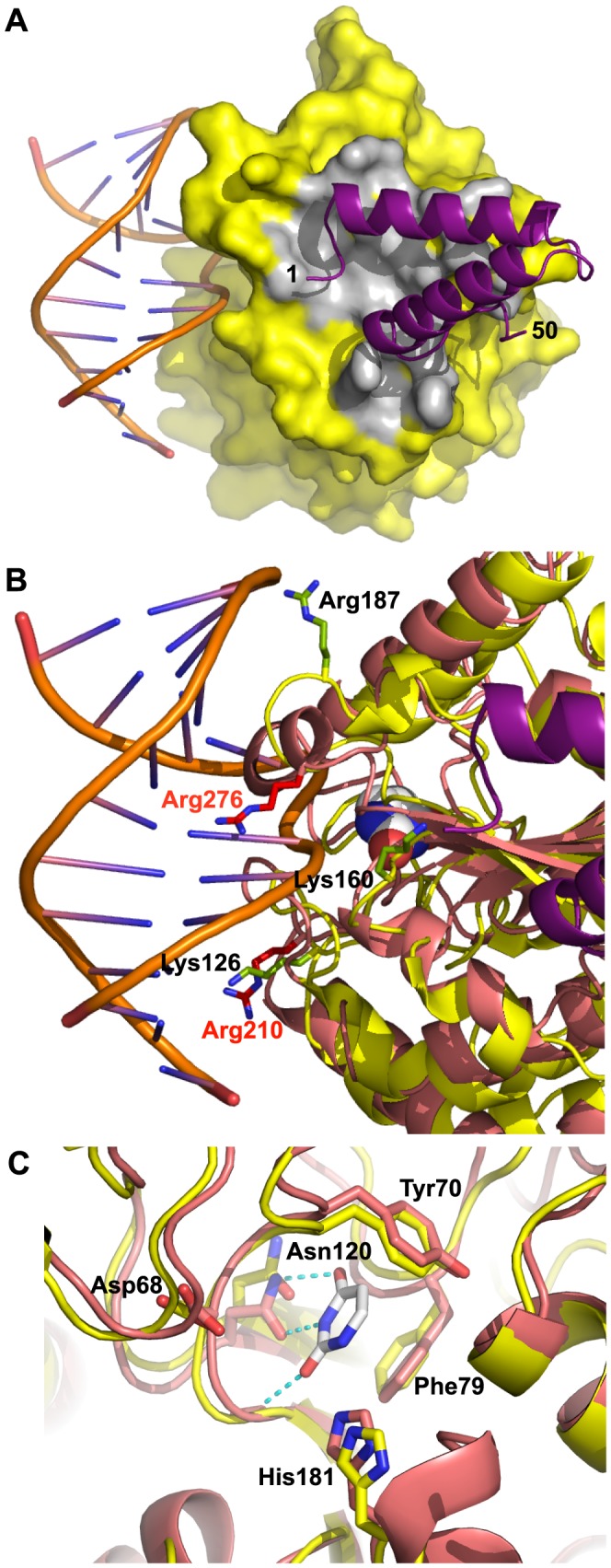
Model of D4/A20_1–50_ heterodimer bound to DNA. D4 from the D4/A20_1–50_ complex was superimposed onto the human UDG from the hUDG/DNA complex structure (pdb entry 1SSP). (A) The surface of D4 from the D4/A20_1–50_ complex is shown in yellow, atoms within 4.5 Å from A20 in grey, A20 as cartoon in violet. The DNA is shown in cartoon representation. (B) Basic residues of D4 (in yellow) mutated in the study of Druck Shudowsky *et al*. [28] and affecting the processivity of the vaccinia virus polymerase holoenzyme are shown in light green and labeled in black. The superposed structure of human UDG is shown in orange-red. Structurally equivalent basic residues are shown in red with red labels. An uracil molecule bound in the uracil binding site of the human enzyme is shown as space filling representation with white carbon atoms. (C) Residues of vaccinia virus and human UDG forming the uracil recognition pocket (Tyr70, Phe79 and Asn120) and required for glycosylase activity (Asp68 and His181) are shown in stick representation. The structure of the human enzyme in complex with dsDNA and uracil (white carbon atoms) is shown with orange-red carbon atoms; corresponding residues of D4 are labeled and shown with yellow carbon atoms. The superposition is based on the shown active site residues. The hydrogen bonds involving the uracil molecule are shown as dotted lines.

VACV D4 shows about 20% sequence identity at the protein level with the human UDG and the overall structures are very similar [29]. In order to obtain a model of the D4/A20_1–50_ complex interacting with DNA, we superimposed D4 onto the human UDG bound to DNA [20] (Figures 6A and 6B). The DNA binding site on D4 is clearly distinct from the D4/A20 interface as the DNA binds on a different side of D4. This is consistent with the fact that D4 exists as a catalytically active enzyme within the DNA polymerase holoenzyme [5]. Sequence alignment of VACV D4 with the related UDGs from herpes simplex virus type 1 (HSV-1) and human identified conserved active-site residues [18], [34]. These are Tyr70, Phe79 and Asn120 which are predicted to form the uracil recognition pocket in addition to Asp68 and His181 that are needed for glycosyl bond cleavage (Figure 6C).

So far, two vaccinia viruses (D*ts*30 and D*ts*27) with temperature sensitive defect in DNA synthesis caused by mutation in the D4R gene have been isolated [24]. The mutation in D4 D*ts*30 leads to a Gly179Arg substitution [34] while the D4 ORF of D*ts*27 contains a Leu110Phe substitution [10]. The Gly179Arg mutation was characterized and presented a default in D4/A20 assembly [10]. The mutated residue is located at the D4/A20 interface where the replacement of Gly179 by a bulky Arg residue right in the center of the interface will certainly weaken the interaction (Figure 2D, arrow). In contrast, the Leu110Phe substitution observed in D*ts*27 did not disturb D4/A20 interaction [10]. The Leu residue is located in the hydrophobic core of the protein, away from the D4/A20 interface and the DNA binding domain of D4 (not shown). Molecular modeling shows that the bulkyer aromatic side chain of Phe cannot be accommodated and will weaken considerably the packing of the hydrophobic core of D4 leading to the temperature-sensitive phenotype.

In a recent study, point mutants were generated in the D4 coding region and tested for their ability to function in processivity and to maintain UDG catalytic activity [28]. Three of these mutants (Lys126Val, Lys160Val and Arg187Val) did not function in processive DNA synthesis but retained binding to A20 and to DNA as well as glycosylase activity. Lys126, Lys160 and Arg187 are shown on a close up view of the DNA/D4/A20_1–50_ model together with structural homolog from the human UDG (Figure 6B). Interestingly, even though the three VACV residues are not directly in contact with DNA, all are located in the vicinity of the double helix and may be involved in DNA binding. It is known that the UDG region close to the DNA shows structural rearrangements upon DNA binding [20]. Thus, it is reasonable to postulate that binding of D4 to DNA may induce some local conformational changes which may bring Lys126, Lys160 and Arg187 in contact with the VACV DNA genome. The loss of these contacts might explain the observed phenotype [28]. In the same study, several residues of the interface have been mutated to Ala: these are residues interacting through hydrogen bonds with A20 (Thr175, Thr176 and Ser194, Figures 2D and 2E). The mutants did not show any phenotype, certainly due to the minor importance of losing a single hydrogen bond to the binding affinity.

Inhibitors of protein-protein interaction have emerged as a new tool to modulate protein functions within various classes of targets [35]. We and others believe that molecules interfering with D4/A20 interaction could be attractive new anti-poxvirus compounds [30], [32], [36]–[38]. In their study, Schormann *et al.* have performed an *in vitro* screen allowing the identification of several molecules interfering with His-D4/MBP-A20_1–100_ [30]. They further showed that the selected compounds exhibited both antiviral activity and binding to D4. In an attempt to determine if some molecules presented in this study could interact with the D4/A20_1–50_ interface, we generated three-dimensional models of D4 in complex with some of these small molecules inhibitors using a molecular docking approach based on the Surflex algorithm [39]. Among the 5 compounds from the study of Schormann *et al* [30] (molecules #1, #6, #9, #12 and #15) that were evaluated by the docking algorithm, several Surflex models generated for compounds #1, #9 and #15 reproduced key contact features identified in the D4/A20_1–50_ complex (Figure S1). Interestingly, all these inhibitors contain a hydrophobic phenyl ring derivative (methylphenyl for compound #1, bromophenyl for #9 and fluorophenyl for #15) that is predicted in these models to mimic the steric complementarity tongue/groove interaction between the A20 Trp43 and D4 Arg167 and Pro173. Further experiments with the above mentioned compounds will be necessary to verify the validity of the proposed models. Future work will allow lead optimization and/or *de novo* design of small molecule inhibitors from the D4/A20_1–50_ structure.

## Materials and Methods

### Construction of plasmids expressing His-D4 and His-D4/MBP-A20_1–50_

Full length D4R gene from VACV (Copenhagen strain) was amplified from viral genome using primers 1 and 2 or primers 3 and 4 (Table 2). The PCR fragments were digested and ligated either into the cleaved pPROEX HTb vector (Life Technologies) or into the cleaved pETDuet-1 plasmid (Novagen), for single or co-expression purpose, respectively (Table 2).

**Table 2:** Primers used for cloning.

Constructs		Primers (5′-3′)
pPROEX-D4R	12	CGGGATCCATGAATTCAGTGACTGTATCAC CCCAAGCTTTTAATAAATAAACCCTTGAGCCC
pETDuet-D4R	34	CGGGATCCGATGAATTCAGTGACTGTATCAC ATAAGAATGCGGCCGCTTAATAAATAAACCCTTGAGCCC
pETM-40-A20R_1–50_T2A	56	CATGCCATGGCTTCTAGCGCTGATTTAAC CCCAAGCTTTCATACCTTTTGCACGCC
pETDuet-D4R-A20R_1–50_T2A	78	GGAATTCCATATGAAAATCGAAGAAGGTAAACTGG CCGCTCGAGTCATACCTTTTGCACGCC
pETDuet-D4R-A20R_1–50_WT	910	pACTTCTAGCGCTGATTTAACTpCATGGCGCCCTGAAAATAAAG
pETDuet-D4R-A20R_1–50_W43A	1112	pGCGAAAATAGGCGTGCAAAAGGpGTAAGTAGATGTTCCCCATTC
pETDuet-D4R-R167A-A20R_1–50_WT	1314	pGCGGCAAAGTTAGAATCCCCGGpTATATTCGAGAAATCTGTTTTAC
pETDuet-D4R-P173G-A20R_1–50_WT	1516	pGGGGTAACTACCATAGTGGGpGGATTCTAACTTTGCCCGTAT

DNA encoding the first 50 amino acids of VACV A20 (Copenhagen strain) was PCR-amplified from viral genome with primers 5 and 6 (Table 2) and introduced into the pETM-40 vector (EMBL), downstream of the maltose binding protein (MBP) gene. The sequence encoding the MBP/A20_1–50_ fusion protein was then amplified with primers 7 and 8 and ligated into the digested pETDuet-1 plasmid carrying the VACV D4R gene. Due to the *Nco*I restriction site used to clone the A20 DNA fragment into the pETM-40 plasmid, a Thr to Ala mutation was introduced in A20_1–50_ at position 2 (T2A). In order to express the WT A20_1–50_ peptide, the pETDuet-D4R-A20R_1–50_T2A construct was PCR-amplified using the Phusion Site-Directed Mutagenesis protocol (Thermo Scientific) and phosphorylated primers 9 and 10 (Table 2). The D4/A20_1–50_ mutants described in this study were all engineered using the same protocol starting with the construct pETDuet-D4R/A20R_1–50_WT and the phosphorylated primers 11–16 shown in Table 2. The DNA sequence of each construct was verified by automated DNA sequencing.

### Expression and purification of His-D4/MBP-A20_1–50_ and His-D4

The construct pETDuet-D4R/A20R_1–50_ allows the expression of a non-cleavable N-terminal His-tagged D4 together with A20_1–50_ fused to the C-terminus of the maltose binding protein (MBP), downstream of a TEV protease cleavage site. The recombinant pETDuet-D4R/A20R_1–50_ was transformed into *Escherichia coli* BL21(DE3) strain. An isolated colony was inoculated into LB medium containing carbenicillin (50 µg.mL^−1^), overnight at 37°C. The culture was diluted to 1/1000^th^ into LB medium supplemented with carbenicillin and bacteria were grown until OD_600_ reached 0.4–0.6. The culture was then transferred to 18°C for 30 min before induction of protein expression with 0.1 mM of isopropyl *β*-D-1-thiogalactopyranoside. Bacterial growth was pursued for an additional 16-hour period at 18°C. The culture was harvested by centrifugation and the bacterial pellet was suspended in the following buffer: 50 mM Tris-HCl pH 7.5, 100 mM NaCl, 10 mM Imidazole, 5 mM β-mercaptoethanol and cOmplete, EDTA-free protease inhibitor cocktail (Roche). Bacteria were lysed by sonication (500 ms pulse at 300 W during 5 min at 4°C) and the supernatant was recovered after centrifugation at ∼40,000 g for 30 min at 4°C. Proteins were then loaded onto a 5 mL HisTrap HP column (GE Healthcare) equilibrated with 50 mM Tris-HCl pH 7.5, 100 mM NaCl, 10 mM imidazole. The column was washed with equilibration buffer and proteins were eluted with the same buffer containing 200 mM imidazole. Fractions containing the His-D4/MBP-A20_1–50_ complex were pooled, desalted on a PD10 column (GE Healthcare) in buffer containing 50 mM Tris-HCl pH 7.5, 100 mM NaCl and treated with Tobacco Etch Virus (TEV) protease at a ratio of 1/100 (w/w), during 16 h at 20°C. The His-D4/A20_1–50_ complex was loaded again onto a 5 mL HisTrap HP column (GE Healthcare) and eluted as described above. Proteins were further purified on a size exclusion chromatography (Superdex 75 10/300 GL, GE Healthcare) equilibrated in 50 mM Tris-HCl pH 7.5, 100 mM NaCl. His-D4/A20_1–50_ was concentrated to 8 mg.mL^−1^ prior to crystallization trials. His-D4/MBP-A20_1–50_ mutants described in this report were all purified as the wild type complex.

To express His-D4, *E. coli* Rosetta (DE3)pLysS strain (Novagen) was transformed with the pPROEX-D4R vector. This vector allows the expression of a TEV-cleavable N-terminal hexa-histidine tagged version of D4. Protein expression was essentially performed as for His-D4/MBP-A20_1–50_ except that the culture was grown in the presence of carbenicillin (50 µg.mL^−1^) and chloramphenicol (34 µg.mL^−1^). Bacteria were suspended in a buffer: 25 mM Tris-HCl pH 7.5, 300 mM NaCl, 20 mM imidazole, 5 mM β-mercaptoethanol and cOmplete, EDTA-free protease inhibitor cocktail (Roche) and were lysed by three cycles of freezing and thawing followed by sonication (500 ms pulse at 300 W during 5 min at 4°C). Cleared cell lysate (obtained after centrifugation at ∼40,000 g for 30 min at 4°C) was loaded onto a 5 mL HisTrap HP column (GE Healthcare) and His-D4 was eluted using a 20–200 mM imidazole gradient. The purified protein was desalted on a PD10 column (GE Healthcare) in buffer containing 25 mM Tris-HCl pH 7.5, 300 mM NaCl. The His-tag was subsequently cleaved by the TEV protease as described above. Recombinant D4 was recovered from the flow through fraction of a HisTrap HP column (GE Healthcare) and further purified by size exclusion chromatography (Superdex 75 10/300 GL, GE Healthcare) equilibrated in 25 mM Tris-HCl pH 7.5, 100 mM NaCl. Purified proteins were analyzed on SDS-PAGE and stained with InstantBlue (Expedeon).

### SEC (Size exclusion chromatography)-MALLS (multi-angle laser light scattering) experiments

SEC was performed with a Superdex 75 10/300 GL (GE Healthcare) equilibrated in 50 mM Tris-HCl pH 7.5, 100 mM NaCl. Separations were performed at 20°C with a flow rate of 0.5 mL.min^−1^. 50 µL of a protein solution at a concentration of 10 mg.mL^−1^ were injected. On-line MALLS detection was performed with a DAWN-EOS detector (Wyatt Technology Corp., Santa Barbara, CA) using a laser emitting at 690 nm. Protein concentration was measured on-line by refractive index measurements using a RI2000 detector (Schambeck SFD) and a refractive index increment dn/dc = 0.185 mL.g^−1^. Data were analyzed and weight-averaged molecular masses (Mw) were calculated using the software ASTRA V (Wyatt Technology Corp., Santa Barbara, CA) as described previously [40].

### Thermal shift assay

Experiments were performed in 96-well non-skirted PCR plates (Thermo Scientific). Each 20 µL reactions were carried out in 50 mM Tris-HCl pH 7.5, 100 mM NaCl containing 1 µM D4 or His-D4/A20_1–50_ and 5× Sypro Orange (Molecular Probes). Plates were closed with Microseal B Adhesive Seal (BioRad) and placed into a Mx3005P qPCR system (Stratagene). A temperature increment of 1°C.min^−1^ was applied from 25 to 75°C. Temperature-induced protein unfolding was monitored by measuring the fluorescence signal at 570 nm (with excitation at 472 nm) and data were processed using the MxPro software. Denaturation curves were normalized and the inflection points were used to determine the melting temperature (*T_m_*). Experiments were performed in triplicate and repeated at least twice.

### Crystallization and data collection

His-D4/A20_1–50_ crystals were initially grown at the High-throughput crystallisation laboratory (HTX Lab, EMBL, Grenoble) using the sitting-drop vapour-diffusion technique as previously described [41]. The complex crystallized at 20°C in Grid Screen Ammonium Sulfate (Hampton Research) condition B6: 0.1 M Bicine pH 9, 1.6 M ammonium sulfate. Conditions were manually optimized and the best crystals were observed in 0.1 M Bicine pH 8.7, 1.5 M ammonium sulfate with a 1.5 µL∶1.5 µL protein∶reservoir drop ratio.

Prior to data-collection, crystals were successively transferred in 10% (v/v) and 20% (v/v) glycerol/reservoir cryo-solution before flash-cooling in liquid nitrogen. Data sets were collected from crystals on beamlines ID23-1 and ID14-4 at the European Synchrotron Radiation Facility (ESRF, Grenoble, France). Data were processed with iMOSFLM [42], and scaled with the program SCALA from the CCP4 suite [43].

### Phase determination, refinement and structure analysis

The structure was solved by molecular replacement using a D4 monomer from pdb file 2OWQ [29] as a search model in PHASER [44]. Clear extra density corresponding to A20_1–50_ was readily visible. The model was manually modified using COOT [45] and refined using REFMAC5 [46]. Refinement statistics and model composition are shown in Table 1. Structure superimposition was performed using CCP4MG [47], protein-protein interactions were analysed with PISA [31] and visually checked using PYMOL (The PyMOL Molecular Graphics System, Version 1.4.1 Schrödinger, LLC). Detailed analysis of monomer orientation within the dimers was performed using LSQKAB [48]. All structure-related figures were generated with PYMOL.

### Molecular docking

Compounds #1, #6, #9, #12 and #15 from Schormann *et al* [30] were drawn and cleaned up using the SYBYL 2.0 sketch module (Tripos, Inc.). Partial charges were computed using the Gasteiger-Marsili algorithm as implemented in Sybyl and final 3D compound structures suitable for further molecular modeling studies were generated by applying a final quick energy minimization step (MMF94 force field, 500 iterations and using Sybyl default parameters). Compounds #1, #6 and #15 contain an asymmetric carbon and the resulting stereoisomers were also drawn and treated using the same protocol. Protein/compound complexes models were generated using Surflex 2.6 [39] as implemented in SYBYL-X 2.0. D4 target protein structure was extracted from the His-D4/A20_1–50_ complex X-ray structure and prepared using Sybyl structure preparation tool (default parameters). The docking area entered as Surflex input parameter to generate the protomol was defined by visual investigation of D4/A20 interface and by selecting D4 residues 61, 63, 99, 148, 149, 151–157, 159, 160, 161, 163, 164, 166–168, 170, 172–181, 184, 188–207 and 209. The protomol was automatically generated after setting the threshold and bloat values to 0.5 and 0. For every compound, 20 docking poses were generated and clustered by families according to the docking zone selected at the surface of D4. Docking poses were visually inspected to identify similarities with D4/A20_1–50_ interaction.

## Supporting Information

Figure S1 Docking of small-molecule inhibitors onto the D4 surface. Compounds #1, #9 and #15 are shown in ball-and-stick representation. D4 is presented as a yellow surface. The structure formula of each compound is also given and its hydrophobic phenyl ring derivative involved in the interaction with Arg167 and Pro173 is highlighted by a red box.
(TIF)
