# Supplementary figures and images for: Crystal Structure of the Vaccinia Virus DNA Polymerase Holoenzyme Subunit D4 in Complex with the A20 N-Terminal Domain
Source: PLoS Pathog. 2014 Mar 6;10(3):e1003978. doi: 10.1371/journal.ppat.1003978 (PMC3946371; doi:10.1371/journal.ppat.1003978)

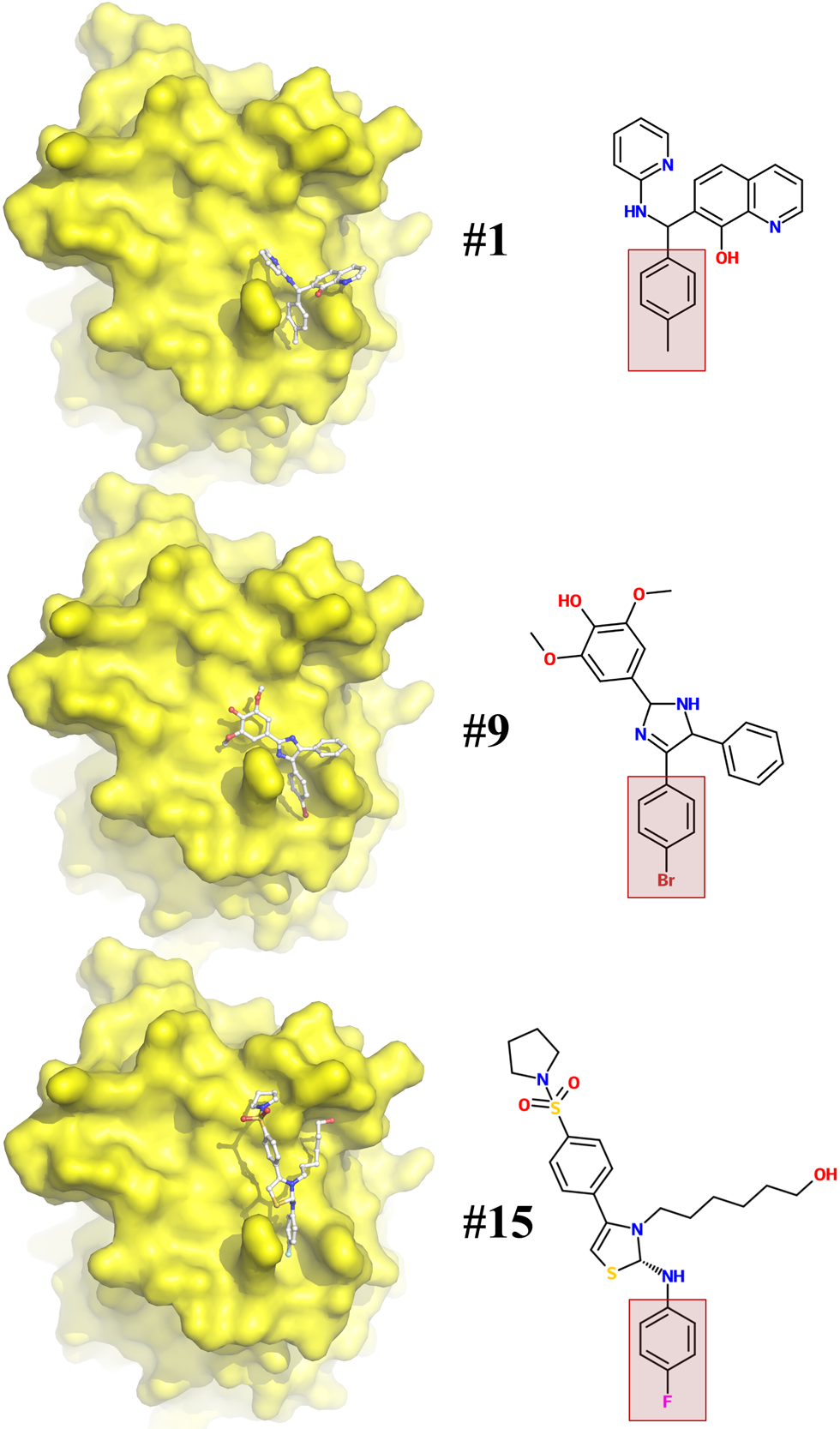

Supplement: Figure S1 — Docking of small-molecule inhibitors onto the D4 surface. Compounds #1, #9 and #15 are shown in ball-and-stick representation. D4 is presented as a yellow surface. The structure formula of each compound is also given and its hydrophobic phenyl ring derivative involved in the interaction with Arg167 and Pro173 is highlighted by a red box. (TIF) [file ppat.1003978.s001.tif]
